# Molecular and phenotypic characteristics of Bardet-Biedl syndrome in Chinese patients

**DOI:** 10.1186/s13023-024-03150-9

**Published:** 2024-04-08

**Authors:** Shiyang Gao, Qianwen Zhang, Yu Ding, Libo Wang, Zhiying Li, Feihan Hu, Ru-en Yao, Tingting Yu, Guoying Chang, Xiumin Wang

**Affiliations:** 1 Department of Endocrinology, Metabolism and Genetics, Shanghai Children’s Medical Center, Shanghai Jiao Tong University School of Medicine, Shanghai, 200127 China; 2 Department of Genetic Molecular Diagnostic Laboratory, Shanghai Children’s Medical Center, Shanghai Jiao Tong University School of Medicine, Shanghai, 200127 China

**Keywords:** Bardet-Biedl syndrome (BBS), Rare disease, Next-generation sequencing, Gene variation, Genotype–phenotype correlation

## Abstract

**Background:**

Bardet-Biedl syndrome (BBS) is a type of non-motile ciliopathy. To date, 26 genes have been reported to be associated with BBS. However, BBS is genetically heterogeneous, with significant clinical overlap with other ciliopathies, which complicates diagnosis. Disability and mortality rates are high in BBS patients; therefore, it is urgent to improve our understanding of BBS. Thus, our study aimed to describe the genotypic and phenotypic spectra of BBS in China and to elucidate genotype–phenotype correlations.

**Methods:**

Twenty Chinese patients diagnosed with BBS were enrolled in this study. We compared the phenotypes of Chinese BBS patients in this study with those from other countries to analyze the phenotypic differences across patients worldwide. In addition, genotype–phenotype correlations were described for our cohort. We also summarized all previously reported cases of BBS in Chinese patients (71 patients) and identified common and specific genetic variants in the Chinese population.

**Results:**

Twenty-eight variants, of which 10 are novel, in 5 different BBS-associated genes were identified in 20 Chinese BBS patients. By comparing the phenotypes of BBSome-coding genes (*BBS2,7,9*) with those of chaperonin-coding genes (*BBS10,12*), we found that patients with mutations in *BBS10* and *12* had an earlier age of onset (1.10 Vs. 2.20, *p* < 0.01) and diagnosis (4.64 Vs. 13.17, *p* < 0.01), whereas patients with mutations in *BBS2*, *7*, and *9* had a higher body mass index (28.35 Vs. 24.21, *p* < 0.05) and more vision problems (*p* < 0.05). Furthermore, in 91 Chinese BBS patients, mutations were predominant in *BBS2* (28.89%) and *BBS7* (15.56%), and the most frequent variants were in *BBS2*: c.534 + 1G > T (10/182 alleles) and *BBS7*: c.1002delT (7/182 alleles), marking a difference from the genotypic spectra of BBS reported abroad.

**Conclusions:**

We recruited 20 Chinese patients with BBS for genetic and phenotypic analyses, and identified common clinical manifestations, pathogenic genes, and variants. We also described the phenotypic differences across patients worldwide and among different *BBS*-associated genes. This study involved the largest cohort of Chinese patients with BBS, and provides new insights into the distinctive clinical features of specific pathogenic variants.

**Supplementary Information:**

The online version contains supplementary material available at 10.1186/s13023-024-03150-9.

## Background

Bardet-Biedl syndrome (BBS, OMIM 209900) is a rare multisystemic non-motile ciliopathy that is usually inherited in an autosomal recessive pattern. In some families, oligogenic inheritance and modifier gene variants for BBS have also been suggested [1, 2]. In the general population, BBS has a prevalence of 0.7/100,000 [3]. However, the incidence of BBS is varies as follow: 1 in 160,000 in North America and Switzerland; 1 in 17,000 in Kuwait—Bedouin populations; 1 in 13,500 in Newfoundland and the Middle East; 1 in 6900 in Jahra district; and 1 in 3,700 individuals in the Faroe Islands [4–9]. Currently, no data exist on the prevalence of this disease in China. More than 100 cases of BBS in Chinese patients have been previously reported in single cases and cohort studies; however, less than 50% of these cases were genetically confirmed [10]. The major clinical features of BBS include: retinal degeneration, obesity, polydactyly, cognitive impairment, hypogonadism and renal dysfunction [11]. Retinal disease is the most common feature, affecting almost all BBS patients [12]. Early retinal degeneration typically presents first as night blindness, followed by a progressive decline in vision, and eventually an overall loss of vision [13]. Patients with BBS usually have a normal birth weight, followed by rapid weight gain, and often develop obesity within 3 years of age, along with bulimia. Polydactyly can be observed at birth and mostly postaxial polydactyly. The renal phenotype of BBS is varies highly and can include structural and functional abnormalities.Urologic abnormalities, such as hydronephrosis and vesicoureteral reflux, with urinary concentration defects (symptoms of polyuria and polydipsia) and developmental anomalies, including horseshoe, ectopic, duplex, or absent kidneys, are common in patients with BBS, these abnormalities frequently lead to renal insufficiency, which is the main cause of death in patients with BBS. Secondary features include congenital heart disease, metabolic abnormalities, developmental delay, dental anomalies, anosmia and hearing loss [11]. Clinical diagnosis of BBS is based on the presence of at least four primary features or a combination of three primary plus two secondary features [14].

Owing to the phenotypic heterogeneity of BBS, it is easy to miss a diagnosis based on clinical manifestations and laboratory examinations alone, therefore, molecular genetic testing is required. Twenty-six genes have been reported to be involved in BBS to date: *BBS1*-*BBS21*, *IFT74*, *SCLT1*, *SCAPER* and *NPHP1,* all of which are localized in the primary cilia and participate in ciliary composition and function [15]. Most of them encode the subunits of the BBSome multi-subunit complex (*BBS1, 2, 4, 5, 7, 8, 9, 17, 18*), which is localized at the ciliary basal body, and their connection to the cilium in the outer segment of the photoreceptor has been thought to play a role in protein trafficking [16]. The second largest group of patients with BBS have mutations in genes encoding chaperonins, which assisting the assembly of the BBSome (*BBS6,10,12*) [17]. Others localize at the ciliary base or at the centrosome, participating in BBSome recruitment [18]. With the rapid development of next-generation sequencing, the number of case reports of BBS patients is increasing; however, there is a lack of large cohort studies of BBS patients in China, making it difficult to summarize the common characteristics of Chinese BBS patients. Here, we describe the genotypic and phenotypic spectra of 20 Chinese BBS patients, to our knowledge, this is the largest BBS cohort in China. Furthermore, the phenotypes of BBS patients in China were compared to those found in three cohorts from Spain, Germany and India [19–21]. In addition, genotype–phenotype correlations were analyzed. We also assembled all of the variants identified in the Chinese BBS patients (*n* = 91) from our study and previous reports, identified distinct and common variants within Chinese patients.

## Materials and methods

### Patients

**Fig. 1:**
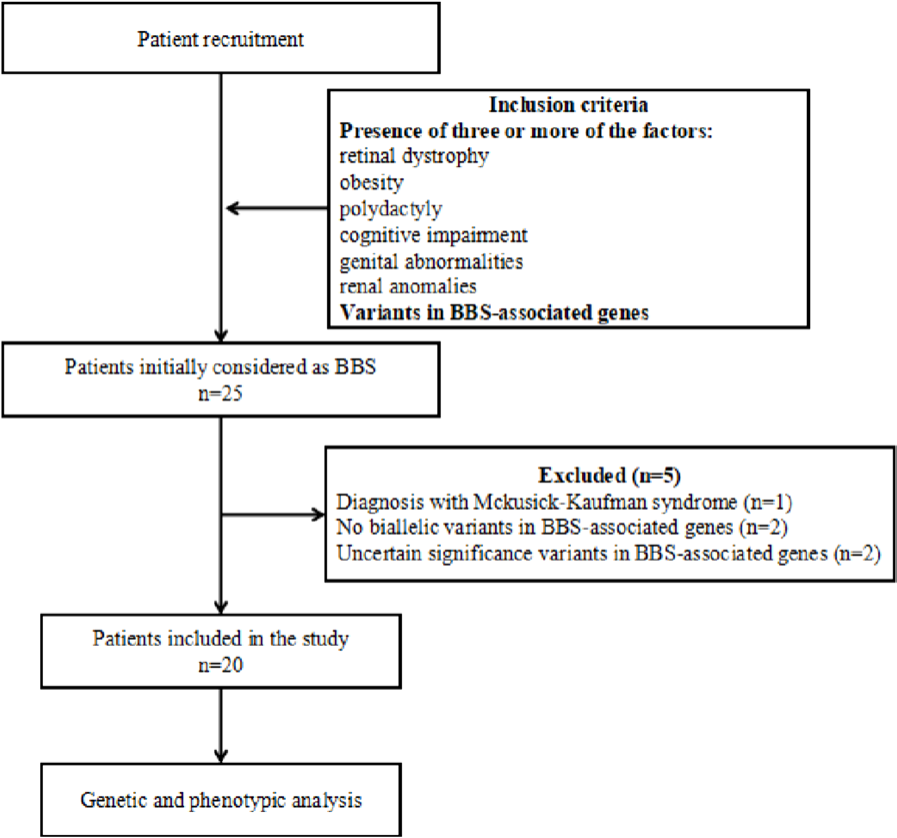
Flowchart of patients recruitment and variants discovery approach. BBS, Bardet-Biedl syndrome

This study was designed as a single-center retrospective evaluation of BBS patients referring to the Department of Endocrinology and Metabolism in Shanghai Children’s Medical Center between 2017 and 2023. Only patients with three or more major clinical manifestations of BBS and variants in BBS-associated gens were included in this study. Patients were excluded if they met one of the following criteria: 1) Diagnosis with Alstrom syndrome, Meckel syndrome, Prader-Willi syndrome, Mckusick-Kaufman syndrome, or Laurence-Moon syndrome; 2) There were no biallelic variants in BBS-associated genes; and 3) Presence of variants of uncertain significance in BBS-associated genes (Fig. 1). Ethical approval for this study was obtained from the ethics committee of Shanghai Children’s Medical Center. Written informed consent was obtained from all the participants or their guardians before information was collected.

### Clinical assessment

Phenotypic data were obtained from these patients, including general information, personal history, medical history, examination reports, and laboratory reports. Obesity was defined as body mass index (BMI) ≥ 28 kg/m^2^ and overweight was defined as BMI ≥ 24 kg/m^2^ for adults according to the criteria from Working Group on Obesity in China (Pan et al., 2021). For children between 0 and 18 years old, overweight or obesity was defined according to the Chinese reference values released by the Chinese child growth standards (2009 edition)  [22]. The growth of the children was evaluated using the height standard deviation score according to the Chinese child growth standards (2009 edition)  [22], while, some parameters, such as cognitive impairment, olfactory and hearing abnormalities, were subjectively evaluated.

### Genetic sequencing

Peripheral blood samples were collected from patients and their parents (F14, F17-I, F17-II, F18-I and F18-II) after informed consent was obtained. Genomic DNA was isolated from peripheral blood leukocytes using the Gentra Puregene Blood Kit (Qiagen). Targeted next generation sequencing (NGS) was performed using a capture panel including 196 known BBS genes and inherited retinal disease (IRD) genes (Supplementary Table S1). Sequencing was performed and clusters were generated with an Illumina HiSeq 2000 system (Illumina, Inc.) and an Illumina cBot system (Illumina Inc, San Diego, CA, USA) respectively. The original sequencing data were assessed using FastQC (v0.11.9, Babraham Research Institute) for quality control and Fastp (v0.20.1, Visible Genetics Inc.) for data filtering. The sequence reads were aligned to the reference human genome (GRCh37/hg19) using SpeedSeq (v0.1.2, Ira Hall Lab). Variant prioritization was performed to first filter out variants that hada minor allele frequency > 1% against the 1,000 Genomes Project. Then retinal dystrophy, obesity and polydactyly were selected as the filtering clinical symptoms to further analyze those variants. The pathogenicity of missense and synonymous variations were analyzed using ClinVar, HGMD Professional and four software prediction programs: PolyPhen-2 (http://genetics.bwh.harvard.edu/pph2/), SIFT (http://sift.jcvi.org/), PROVEAN (http://provean.jcvi.org/index.php), and MutationTaster (http://mutationtaster.org/ChrPos.html). SpliceAI (https://spliceailookup.broadinstitute.org/) was used to analyze the potential splicing effect of intronic variations. Variants detected were confirmed by Sanger sequencing in the proband and her parents. Next-generation sequencing in other patients were performed through other commercial companies or hospitals. All variants were re-identified by the geneticists at Shanghai Children’s Medical Center according to the guidelines recommended by the American College of Medical Genetics and Genomics (ACMG).

### Statistical analysis

Qualitative data are expressed as frequency (%) and compared using Chi-squared test or Fisher exact test, when more than 20% of the theoretical frequency is less than five or the theoretical frequency is 0. Quantitative data showed as mean ± SD and comparisons were performed by non parametric tests: Kruskal‐Wallis test. SPSS 25.0 (Statistical Package for the Social Sciences Inc., Chicago, IL, United States) was used for statistical analyses. *P* < 0.05 was considered statistically significant with two-sides.

## Results

### Demographic data

**Fig. 2:**
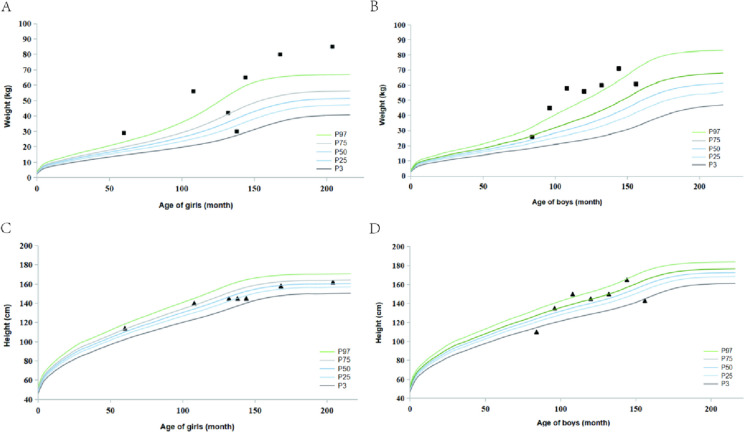
Basic characteristics of the Chinese cohort. Weight of girls (**A**) and boys (**B**), and height of girls (**C**) and boys (**D**) included in this study. Each point represents a patient, and the standard child weight and height curves are extracted from the Chinese child growth standards (2009 edition) [12]

We initially recruited 25 patients who were then subjected to a careful check of medical records, resulting in the exclusion of five patients. This left us with 20 patients from 18 non-consanguineous families (9 females and 11 males). Family 17 and 18 each has two siblings. Among them, three patients (F2, 3, 15) have been previously reported [23–25]. Most of the patients were from eastern China with a mean age of 16.0 ± 9.08 years old (range 5–34 years). Referring to the obesity criteria for children aged 0–18 years [22], the age-weight distribution of BBS patients was drawn separately by sex (Fig. 2A, B), 17 patients were overweight or obesity, with no difference between males and females. The height of the patients was close to the normal range, according to the Chinese Growth Reference [22] (Fig. 2C, D).

### Genetic results

**Fig. 3:**
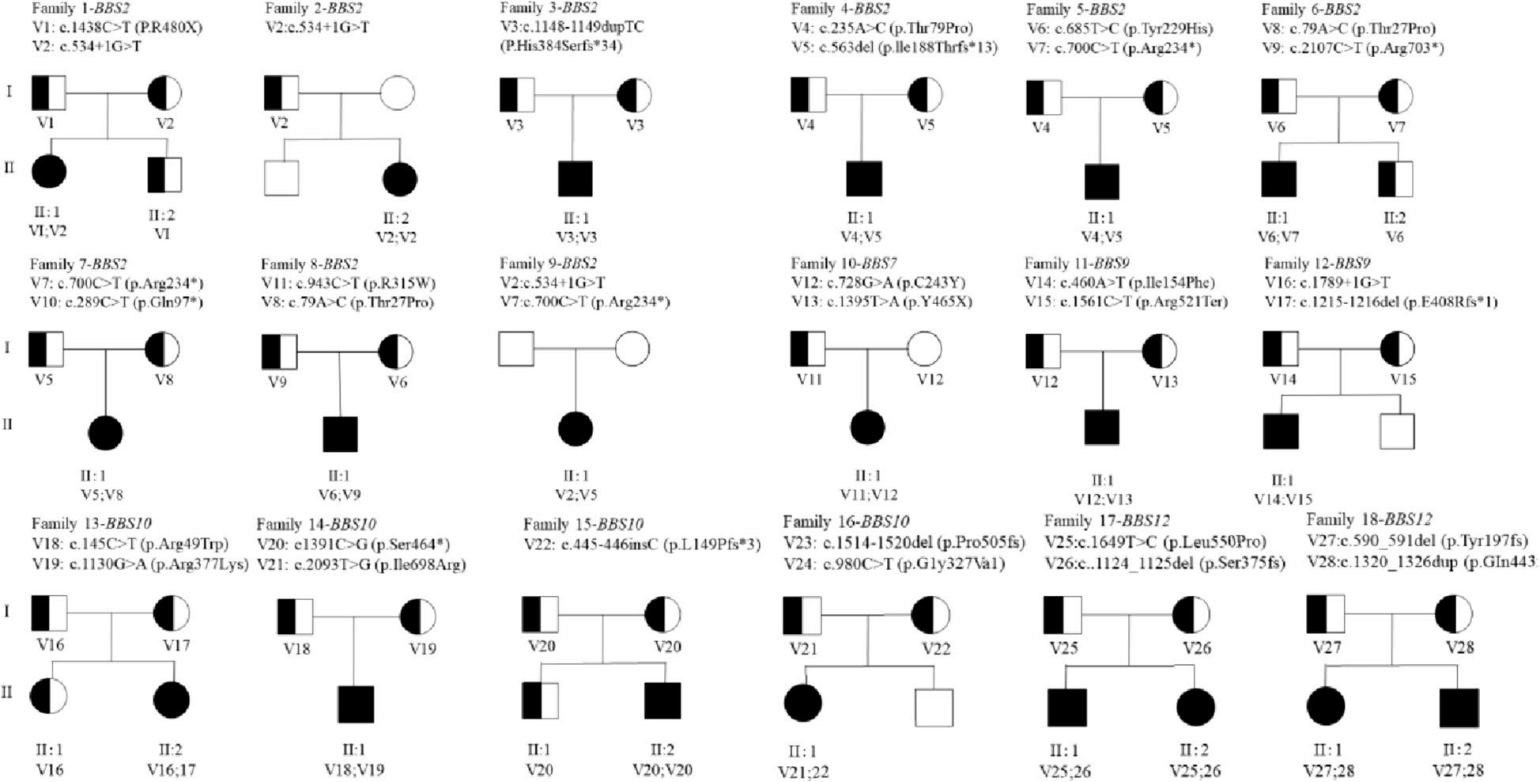
Pedigrees of BBS families. The squares and circles indicate men and women, respectively. The black solid square or circle represents the affected individual. The half black solid circle or square represents the variation carrier

Using exome sequencing, we identified 28 different disease-causing variants in five different *BBS* genes across the 20 patients (Table 1). Nine patients (45%) harbored biallelic (apparent homozygous or two heterozygous) variants in the *BBS2* gene, one (5%) in the *BBS7* gene, two (10%) in the *BBS9* gene, four (20%) in the *BBS10* gene, and four (20%) in the *BBS12* gene. Missense variants were the most common type of mutation (42.9%), followed by frame-shifting small insertion, deletion and duplication mutations (28.6%), nonsense mutations (21.4%) and variants likely resulting in mis-splicing (7.1%). These variants were confirmed to completely cosegregate with the disease in the family in which they occurred, based on the recessive mode of inheritance identified by Sanger sequencing (Fig. 3). Eighteen of the 28 variants in the five *BBS* genes have been reported, while the 10 variants have not been included in the ClinVar or Human Gene Mutation Database Professional to date; therefore, they are considered novel (*BBS10*, 38.46%; *BBS9*, 23.08%; *BBS12*, 23.08%; *BBS2*, 7.69%; *BBS7*, 7.69%). The allele frequencies of these variants were < 0.1% against the 1,000 Genomes Project and the Exome Aggregation Consortium databases. All 28 variants were classified as pathogenic or likely pathogenic according to the ACMG guidelines. In this cohort, *BBS2*: c.534 + 1G > T was detected in three non-consanguineous families (F1, F2 and F9), and among the pathogenic *BBS* genes in 20 patients, *BBS2* was the most common (45%).

**Table 1:** Genotypic spectra of BBS patients in this study

Patient	Gene	Exon/Intron	Nucleotide change	Protein change	Hom/Het	gnomAD	Report	ACMG
F1	*BBS2*	Exon12 Intron4	c.1438C > T c.534 + 1G > T	p.R480*	het	0.000024 0.0001025	Janssen et al. (2011) [26] Wang et al. (2013) [27]	P(PVS1,PS4,PM1,PM2) P(PVS1 + PM1 + PM2)
F2	*BBS2*	Intron4	c.534 + 1G > T		hom	0.0001025	Wang et al. (2013) [27]	P(PVS1 + PM1 + PM2)
F3	*BBS2*	Exon10	c.1148_1149dupTC	p.His384Serfs*34	hom	-	Chen et al. (2017) [23]	P(PVS1 + PM2 + PP4)
F4	*BBS2*	Exon5 Exon2	c.563del c.235A > C	p.lle188Thrfs*13 p.Thr79Pro	het het	0.0002 -	Xing et al. (2014) [28] Nykamp et al. (2017) [29]	P(PVS1,PS4,PM2) LP(PS4,PM2,PM3,PP4)
F5	*BBS2*	Exon6 Exon6	c.700C > T c.685 T > C	p.Arg234* p.Tyr229His	het het	0.000027 0.000025	Nishimura et al. (2001) [30] Gao et al. (2019) [31]	P(PVS1 + PM2 + PP4) LP PM2,PM3,PP3,PP4)
F6	*BBS2*	Exon17 Exon1	c.2107C > T c.79A > C	p.Arg703* p.Thr27Pro	het het	- -	Xu et al. (2015) [32] Meng et al. (2021) [33]	P(PVS1,PM2,PP4) LP(PS4PM2,PM3,PP4,PP3)
F7	*BBS2*	Exon6 Exon2	c.700C > T c.289C > T	p.Arg234* p.Gln97*	het het	0.000027 -	Nishimura et al. (2001) [30] Wang et al. (2013) [27]	P(PVS1 + PM2 + PP4) P(PVS1 + PM2 + PP4)
F8	*BBS2*	Exon9 Exon1	c.943C > T c.79A > C	p.R315W p.T27P	het het	0.000021 -	Katsanis et al. (2001) [34] Meng et al. (2021) [35]	LP(PS4PM1,PM2 + PP2 + PP3) LP(PS4PM2,PM3,PP4,PP3)
F9	*BBS2*	Exon6 Intron4	c.700C > T c.534 + 1G > T	p.Arg234*	het het	0.000027 0.001025	Nishimura et al. (2001) [30] Wang et al. (2013) [27]	P(PVS1 + PM2 + PP4) P(PVS1 + PM2 + PP4)
F10	*BBS7*	Exon14 Exon8	c.1395 T > A c.728G > A	p.Y465X p.C243Y	het het	- 0.00004	Meng et al. (2021) [33] Shin et al. (2015) [36]	P(PVS1 + PM2 + PP4) LP(PS1,PM2,PM3,PP3)
F11	*BBS9*	Exon6 Exon16	c.460A > T c.1561C > T	p.lle154Phe p.Arg521Ter	het het	- -	Novel Novel	LP(PM2 + PM3,PP3,PP4) P(PVS1 + PM2 + PP4)
F12	*BBS9*	Exon11 Exon17	c.1215_1216del c.1789 + 1G > T	p.E408Rfs*1	het het	- 0.00007957	Novel Baralle et al. (2005) [37]	P(PVS1 + PM2 + PP4) P(PVS1,PM2,PM3,PP4)
F13	*BBS10*	Exon1 Exon2	c.145C > T c.1130G > A	p.Arg49Trp p.Arg377Lys	het het	- -	Stoetze et al. (2006) [38] Novel	P(PS3,PS4PM1,PM2,PP3,PP5) LP(PM2,PM3,PP3,PP4)
F14	*BBS10*	Exon2 Exon2	c.1391C > G c.2093 T > G	p.Ser464* p.Ile698Arg	het het	0.000012 -	Scheidecker et al. (2015) [39] Novel	P(PVS1,PM1,PM2,) LP(PM2,PM3,PP3,PP4)
F15	*BBS10*	Exon2	c.445_446insC	p.L149Pfs*3	hom	-	Lin et al. (2018) [24]	P(PVS1,PM2,PP4)
F16	*BBS10*	Exon2 Exon2	c.1514_1520del c.980C > T	p.Pro505fs p.G1y327Va1	het het	- -	Novel Novel	P(PVS1,PM2,PP4) LP(PM2,PM3,PP3,PP4)
F17-I	*BBS12*	Exon2 Exon2	c.1124_1125del c.1649 T > C	p.Ser375fs p.Leu550Pro	het het	- -	Novel Novel	LP(PVS1,PM2) LP(PM2,PM3,PP3)
F17-II	*BBS12*	Exon2 Exon2	c.1124_1125del c.1649 T > C	p.Ser375fs p.Leu550Pro	het het	- -	Novel Novel	LP(PVS1,PM2) LP(PM2,PM3,PP3)
F18-1	*BBS12*	Exon2 Exon2	c.590_591del c.1320_1326dup	p.Tyr197fs p.GIn443fs	het het	0.000008 -	Novel Dulfer et al. (2010) [40]	P(PVS1,,PM2,PM3,PP4) P(PVS1,PM2,PM3,PP4)
F18-II	*BBS12*	Exon2 Exon2	c.590_591del c.1320_1326dup	p.Tyr197fs p.GIn443fs	het het	0.000008 -	Novel Dulfer et al (2010) [40]	P(PVS1,,PM2,PM3,PP4) P(PVS1,PM2,PM3,PP4)

### Phenotype observations

Detailed clinical manifestations of the patients are presented in Supplementary Table S2. BBS symptoms show variable penetrance. Retinal dystrophy (95%), polydactyly (95%), and being overweight or obese (85%) were the three most common clinical features in our cohort. However, other primary clinical manifestations, such as renal abnormalities and cognitive impairment, were not systematically evaluated or did not manifest temporarily in some patients; therefore, their prevalence may be underestimated. Night blindness or vision decline was the initial symptom in 80% of patients with BBS; however, most of them were misdiagnosed with amblyopia upon their first visit to the hospital. Ninety percent of patients with BBS had visual impairment before 7 years of age, including night blindness, photophobia, nystagmus, and vision field defects, and 95% of them progressed into retinitis pigmentosa, while, blindness was not observed in our cohort, possibly due to the young age. All patients had normal birth weight followed by rapid weight gain, and 70% of them were overweight or obese within 3 years of age. Seventy-five percent of patients had gonadal dysplasia (90.9% of males had micropenis, 44.4% of females had reproductive system anatomical abnormalities, and 11.1% of females had low sex hormone secretion). The prevalence of renal abnormalities was lower than that of other symptoms (22.2%) (22.2% renal structural abnormalities, and 11.1% renal insufficiency). Among the secondary symptoms: 75% of patients with BBS had development delay; and 42.1% of them had orodental anomalies, such as crowding of the teeth, microdontia, and hypodontia. Furthermore, 25% of patients had insulin resistance or diabetes mellitus. Moreover, 20% of patients had liver disease, including nonalcoholic fatty liver disease or elevated alanine aminotransferase levels; Olfactory impairment was relatively rare (10.5%), and no hearing impairment was observed in our cohort. However, they were evaluated subjectively by the patient's family, which needs further verification. What is worth noting is that polydactyly and renal cysts were found in three patients during fetal life by pregnancy examination, but were ignored and not diagnosed with BBS until visual problems occurred. Therefore, in fetuses with polydactyly, genitourinary abnormalities, and/or hydrometrocolpos, BBS should be considered [41].

Furthermore, some non-classical BBS clinical features were documented in our cohort, and most of the symptoms presented only in one patient, including: epilepsy, congenital megacolon, bladder diverticulum, recurrent urinary tract infections, and vaginal atresia; however, the reliable association of these abnormalities with the BBS phenotype spectrum should be further investigated.

### Phenotypic comparison between our Chinese cohort and cohorts from other countries

To understand whether the clinical manifestations of BBS are consistent across patients worldwide, we compared the phenotypes of our Chinese cohort with those of the three cohorts from other countries: Spain, Germany, and India [19–21] (Table 2). The average age in the Chinese cohort was younger than that in the Spanish and German cohorts, but older than that in the Indian cohort. Additionally, the age at diagnosis in our cohort was younger than that in the German patients, which may be due to the rapid development of molecular diagnostic techniques, which has enabled faster diagnosis in the more recent Chinese cohort. The main clinical manifestations of BBS were similar in these cohorts. Visual problem are typical symptoms of BBS, which occurred in almost all patients in the four cohorts. The incidence of obesity or overweight and polydactyly were also high; however, no statistically significant differences were observed among these cohorts. Conversely, the Chinese cohort had a higher prevalence of hypogonadism (a higher incidence of micropenis and small testicles in males) than the German cohort (*p* < 0.01). This discrepancy may be associated with the assessment criteria or ethnic differences. Compared with the other five main clinical features, the incidence of renal abnormalities in the four cohorts was relatively low; some patients in these cohorts did not undergo systematic evaluation of the kidney. Since kidney-related clinical features may gradually appear, which may lead to the incidence of renal dysfunction being underestimated; regular follow-up renal ultrasonography and function tests are recommended. Overall, we observed that the main clinical symptoms of BBS are common in patients worldwide.

**Table 2:** Clinical features of our Chinese cohort compared with Spanish, or German or Indian study

**Features**	**Chinese** **Cohort(*N* = 20)**	**Spanish** [19] **Cohort(*N* = 52)**	**German** [20] **Cohort(*N* = 61)**	**Indian** [21] **Cohort(*N* = 64)**
Age (mean ± SD, y)	16.0 ± 9.08	**39.6 ± 17.86**^a***^	NA	13.4 ± 6.42
Age at diagnostic exam(mean ± SD, y)	8.00 ± 7.22	NA	**24.49 ± 12.30**^a***^	NA
Gender (M/F)	11/9	28/24^b^	35/26^b^	44/20^b^
RP, n/N (%)	19/20 (95)	51/52 (98)^c^	61/61 (100)^c^	62/64(97)^c^
OB, n/N (%)	17/20 (85)	44/50 (88)^c^	48/61 (79)^c^	57/64(89)^c^
PD, n/N (%)	19/20 (95)	41/52 (79)^c^	53/61 (87)^c^	55/64(86)^c^
CI, n/N (%)	10/20 (50)	29/49 (59)^b^	17/61 (28)^b^	42/64(66)^b^
Gonad, n/N (%)	15/20 (75)	17/35 (49)^b^	**6/22(27.3)**^b**^	42/64(66)^b^
RA, n/N (%)	4/18 (22)	12/36 (33)^b^	18/61 (30)^b^	10/64(16)^c^
Development delay	15/20(75)	14/22(64)^b^	**21/61(34)**^b**^	**31/64(48)**^b*^
Diabetes	3/18(16.7)	2/64(3)^c^	5/61(8)^c^	18/64(28)^c^
Hearing disturbances	0/19(0)	**9/52(17)**^c*^	2/61(3)^c^	**14/64(22)**^c*^
neurology	3/20(15)	**14/22(63.6)**^b***^	18/61(29.5)^b^	**29/64(45.3)**^b*^

*F* female, *M* male, *NA* data not available, *RD* retinal dystrophy, *OB* obesity, *PD* polydactyly, *CI* cognitive impairment, *RA* renal abnormalities; bold indicates that there are significant differences between the two groups (*p* < .05)

^a^*p*-value by the Mann–Whitney test

^b^*p*-value by the Pearson Chi-square test

^c^*p*-value by the Fisher exact test, *p < 0.05, **p < 0.01, ***p < 0.001

Regarding the secondary clinical features of BBS, the higher incidence of developmental delay in the Chinese cohort was significantly different from that in the German (*p* < 0.01) and Indian (*p* < 0.05) cohorts. However, the lower incidence of hearing impairment and mental problems in the Chinese cohort than in the others may be due to the differences in patient ages. The average age in the Chinese cohort was 16.0 years while it was 39.6 years in the Spanish cohort. This suggests that the clinical symptoms may become more serious with age. Moreover, atypical BBS features observed in the Chinese cohort, such as congenital Hirschsprung, epilepsy, and hypospadias, appeared in patients with BBS from other countries, suggesting that these symptoms are associated with BBS and the widespread role of primary cilia in the system.

### Genotype-phenotype correlations

There is substantial genetic and clinical heterogeneity in BBS patients; however, the mechanism underlying this variation remains unknown. Clinical phenotypes may vary according to the variant genes; however, there is also a large variability in clinical manifestations among individuals with the same genotype.

**Fig. 4:**
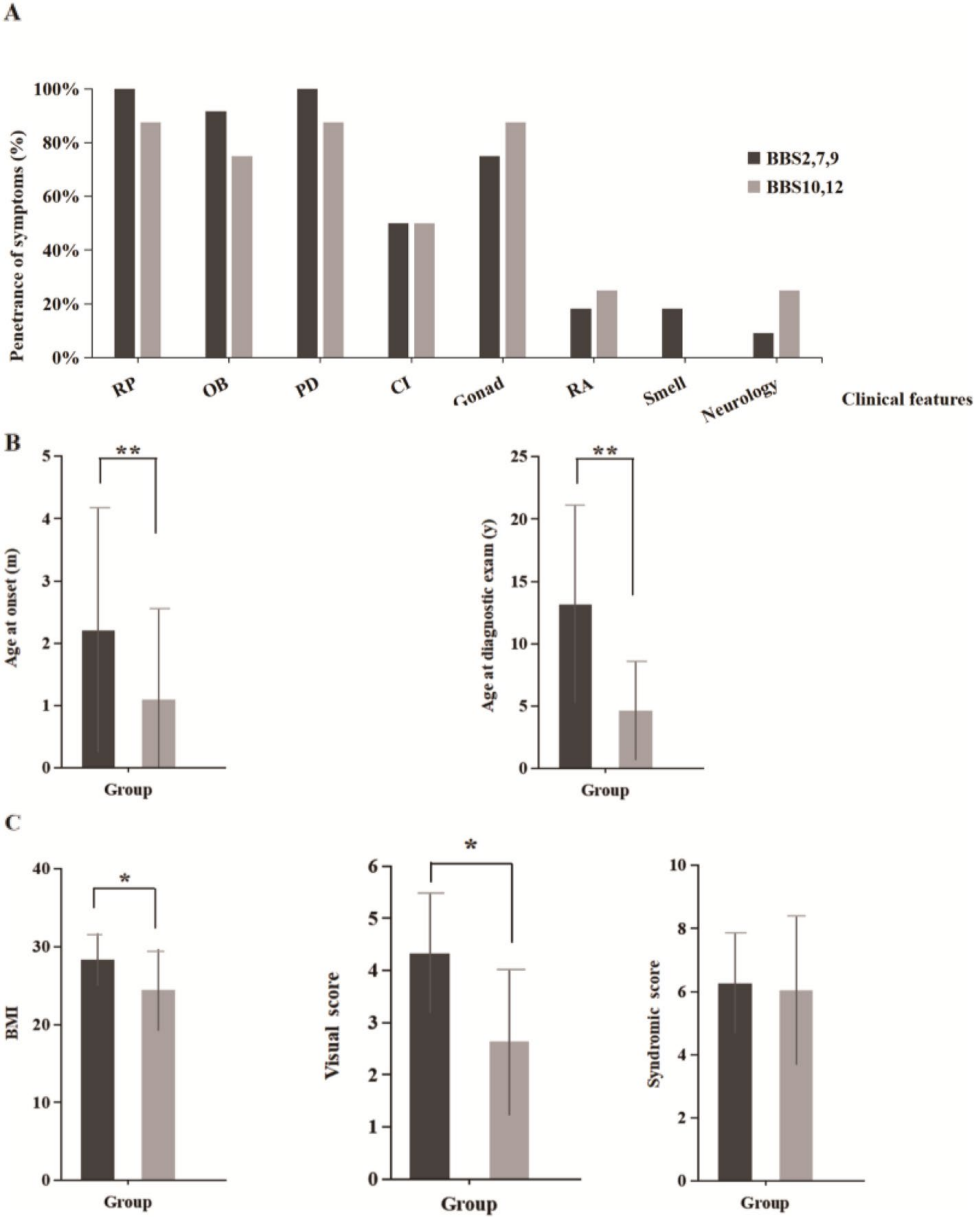
Genotype–phenotype correlations in our cohort of BBS patients. **A** Distribution of primary and secondary features between patients with mutations in BBSome genes and chaperonin-like genes. **B** The age of onset and diagnosis of patients with mutations in *BBS2*,*7*,*9* versus *BBS10*,*12*. Distribution of primary and secondary features between patients with mutations in BBSome genes and chaperonin-like genes. **C** BMI, visual score, and syndromic score in BBS patients with *BBS2,7,9* mutations and *BBS10,12* mutations. *p < 0 .05, **p < 0 .01

To investigate the nature of the clinical and genetic variations in our cohort, the 20 patients were divided into two groups according to the gene in which they carried a BBS-associated variant. The first group carried mutations in proteins of the BBSome complex (BBS2, 7, 9), which is involved in transport in the cilia, while the second group carried mutations in BBSome chaperones. Correlations between the genotype and phenotype were assessed in the two groups by comparing the age at onset and diagnosis, BMI value, incidence of primary and secondary symptoms, visual score (calculated as the number of visual symptoms present) and syndromic score (calculated as the number of major and secondary symptoms present). Genotype–phenotype correlations obtained by statistical analysis are shown in Fig. 4. Compared to BBSome gene mutations, patients with variations in *BBS10, 12* had an earlier age of onset and diagnosis (*P* < 0.01), which may partly reflect their poor prognosis. In addition, patients with BBSome gene variations had higher BMI values (*P* < 0.05), owing to the age difference between the two groups, this may reflect obesity becoming more severe as age increases, and does not fully indicate the difference in obesity between these two groups. Furthermore, patients with variations in *BBS2, 7*, and *9* had higher visual scores ((*P* < 0.05) and more severely impaired vision. However, it is possible that there were fewer patients with BBS in our cohort, statistically significant differences were not observed in the incidence of primary and secondary symptoms, and syndromic score was similiar in these two groups.

### Spectrum of *BBS-related genes* variants in Chinese patients

**Fig. 5:**
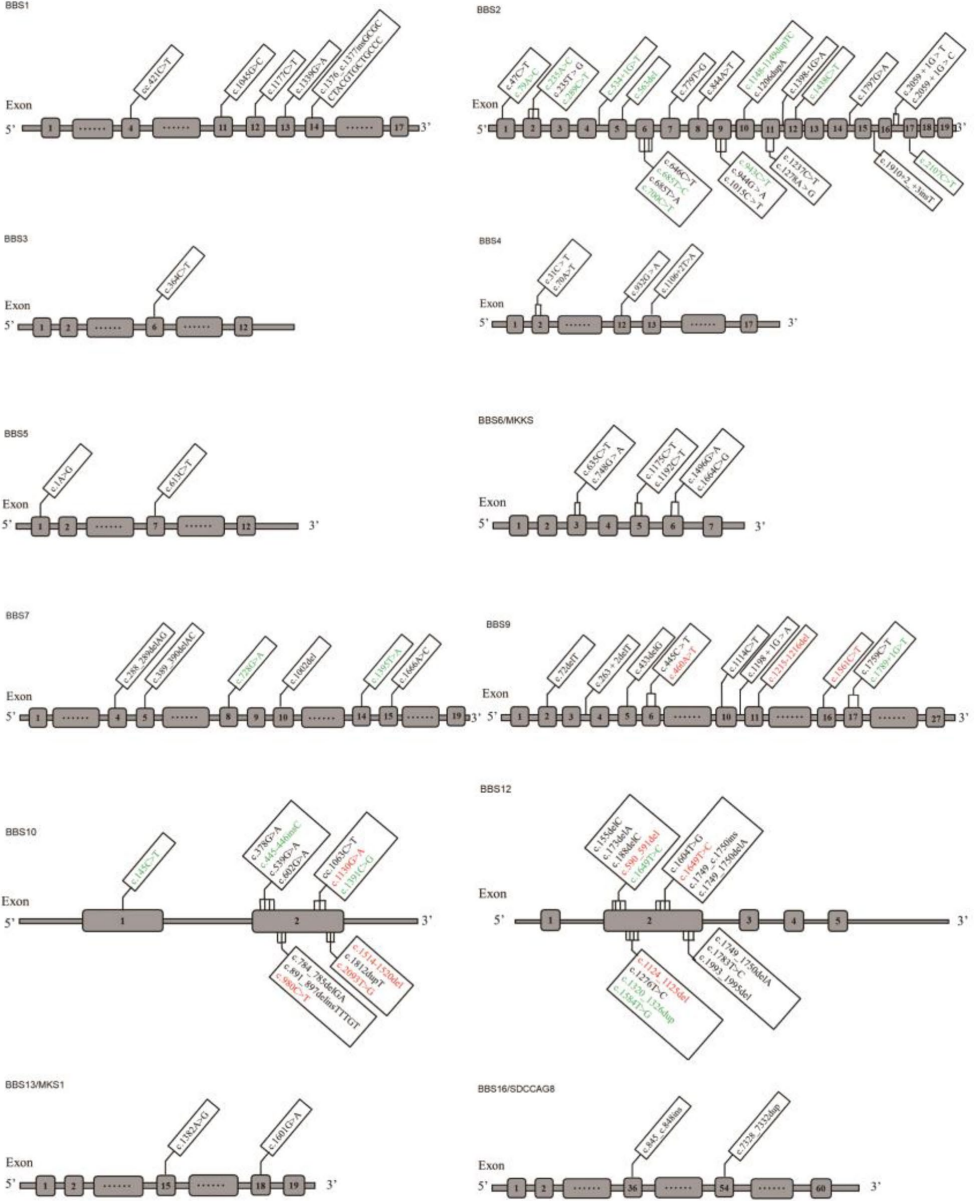
Summary of BBS variants identified in 91 Chinese BBS patients from 87 families. Sixty-eight previously reported variants in Chinese BBS patients are shown in black, and 28 variants were identified in this study, among them 18 varients which have been reported are shown in green and 10 novel variants are shown in red

To define the genetic spectra of BBS in Chinese patients, we summarized all previously reported variants in Chinese BBS patients. To date, 71 Chinese BBS patients from 67 families have been identified, giving a total of 91 patients from 85 families including those in the current cohort. We retrieved all the variants from the 91 Chinese BBS patients and identified 96 variants in 13 *BBS* genes (*BBS1*, *BBS2*, *BBS3*, *BBS4*, *BBS5*, *BBS6*, *BBS7*, *BBS9*, *BBS10*, *BBS12*, *BBS13*, *BBS16*, *CEP290*) (Fig. 5, Supplementary Table S3). The results indicated that *BBS2* (28.89%) and *BBS7* (15.56%) are the hotspot genes in Chinese patients with BBS, with *BBS2*: c.534 + 1G > T (10/182 alleles) and *BBS7*: c.1002delT (7/182 alleles) being the most prevalent variants. A greater variability in mutational load was seen in patients with mutations in *BBS2, BBS10* and *BBS12*, with 27 different mutations identified in *BBS2* and 14 in each of the other two genes. *BBS2* showed more types of variants (ten missense mutations, nine nonsense changes, four deletion, and four truncating frameshift mutations) [22–24, 31, 33, 42–72].

### Genotype comparison of Chinese BBS patients with cohorts from other countries

**Fig. 6:**
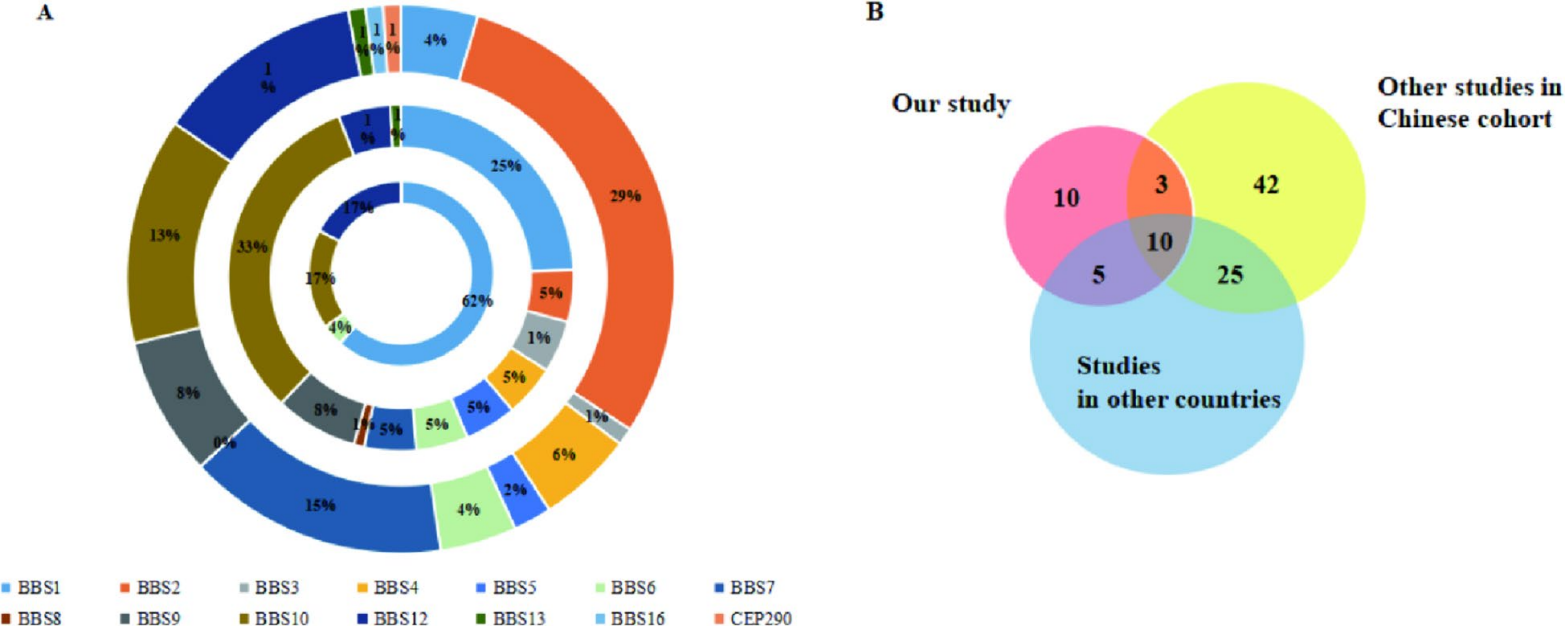
The genotypic spectra of BBS in Chinese and other foreign cohorts. **A** Distribution of genotypes in Chinese patients and other foreign cohorts. The inner circle represents the Spanish cohort, the middle represents the German, and the outer represents the Chinese. The *BBS2* genotype predominates in Chinese patients with BBS followed by mutations in *BBS10* and *BBS2*. *BBS1* and *BBS10* are the most common *BBS* genes in the Spanish and German cohorts. **B** Pie chart for the number of variants identified in our study and other cohorts

Next, We compared the genetic variations in Chinese patients with BBS with cohorts in Germany, Spain and India, aiming to identify the prevalent variants in patients worldwide, and the distinct variants in the Chinese patients. Distribution of these variants in China and other countries is shown in Fig. 6. Some *BBS* genes appeared to have a greater ethnicity-specific frequency than others. This included *BBS1* and *BBS10,* which are the most prevalent *BBS* genes in Spanish and German cohorts, *BBS3* and *BBS10* predominant in Indian cohort, while *BBS2* and *BBS7* are the main pathogenic genes in Chinese patients with BBS. In addition, *BBS2*: c.534 + 1G > T, a frequent variant in Chinese patients with BBS, was not observed in the German cohort. *BBS1*: c.1169 T > G is not a hot spot in Chinese BBS patients as has been described in Spanish cohort.

## Discussion

In this study, 20 Chinese patients with BBS were enrolled for genetic and phenotypic analyses. To the best of our knowledge, this is the largest cohort study of Chinese patients with BBS, and the mean age of the participants was the youngest among all BBS cohorts in China. All patients underwent diagnostic genetic testing and were selected based on the presence of apparent biallelic variants in known *BBS-*associated genes. Some symptoms of BBS are not present at birth, and night blindness or declined vision is generally the first clinical manifestation of the disease; however, patients are frequently misdiagnosed as having amblyopia at the first visit and are diagnosed until vision progressively worsen or other symptoms appear, making the average age at diagnosis relatively late at 8 years.

Although the combination of symptoms in BBS varies, visual impairment, polydactyly, and obesity were the most prevalent clinical manifestations in our and other foreign cohorts, in where the high penetrance of retinal dystrophy appears to be universal among all*BBS* genes tested. Hypogonadism was more prevalent in this study than in previous cohorts from other countries, which may be due to ethnic differences. Moreover, various systemic clinical features, such as congenital Hirschsprung, epilepsy, and hypospadias, have been observed in patients with BBS. Therefore, further exploration of the functions of ciliary proteins is important.

In this study, 28 different variants were found in five different *BBS* genes, of which 10 were novel. Our study, together with other Chinese patient reports, revealed that *BBS2* is a prevalent disease-causing gene among Chinese patients with BBS, and that *BBS2*: c.534 + 1G > T is a hot-spot variant. Mutations such as the M390R in *BBS1* and C91fs*95 in *BBS10* were predominant in European and Caucasian [73], while they were not observed in Chinese patients with BBS. Mutations in *BBS4*, *BBS5* and *BBS8* are most prevalent in the Middle East and North African populations [74]. These results support the hypothesis that different populations have distinct *BBS* mutation profiles.

The links between the phenotype and the genotype among patients with BBS have been addressed by several previous studies in other countries [19, 35], while China lacks a sufficient number of patients with BBS or data analysis limited to one aspect of multiple organ diseases. Here, we systematically analyzed the gene-phenotype correlation in Chinese patients with BBS. Our data suggests that patients with various *BBS* mutations have different prognoses. Patients with *BBS2, BBS7,* and *BBS9* gene variants had more severe visual symptoms and multiple vision problems, such as night blindness, photophobia, decreased vision loss, narrow vision, and nystagmus, suggesting that the function of the BBSome complex is partially independent of chaperones, which is prominent in the development of the retinal dystrophy. Patients with variations in *BBS10* and *BBS12* had an earlier age of onset and diagnosis. which may partly reflect their poor prognosis, this is consistent with previous reports of patients with *BBS10* and *BBS12* having more severe features [75]. A phenomenon that could be explained by BBSome chaperone protein is involved in the early synthesis of BBSome [76]. The penetrance differences of the primary and secondary symptoms, particularly for renal abnormalities, between the patients with variations in genes encoding chaperonin and BBSome complex were not found in either our cohort or the Indian cohort [77], suggesting that BBSome function may be entirely dependent on the chaperonin. Zou Xin-Yi et al. systematically reviewed the patients with BBS reported from China and found that those with variants in *BBS2* had higher hearing impairment, while those with variants in *BBS10* had lower renal abnormality penetrance [41]; Veronika et al. revealed that differences in the penetrance of kidney anomalies among patients make biological sense as the causative genes linked to a high frequency of kidney anomalies, which include *BBS2*, *BBS7*, and *BBS9*, encode structurally similar proteins that form the core of the BBSome [75]. Although this was not observed in our cohort, a comprehensive evaluation of the patients' kidneys should be conducted in the future. Overall, the effects of individual mutations depend on the *BBS* gene carrying the mutation, as well as the extent to which the mutation impairs the function of the gene product.

This study had some limitations. First, because the number of patients enrolled in this study was small, more specific genotype-phenotype relationships could not be analyzed. The rarity of BBS and the lack of wide screening of BBS in China could partly explain the phenomenon. Second, some patients were not systematically evaluated, which may have led to an underestimation of the incidence of BBS features.Additionally, due to the different phenotypes selected by various researchers, only the co-observed phenotypes between our cohort and others worldwide were compared; therefore, many different phenotypes may be missed. Furthermore, all variants of *BBS* in our cohort were classified as pathogenic or likely pathogenic according to the ACMG, so functional studies were not performed. More reaearch should be conducted to explore genotype-phenotype correlations and specific mechanisms.

## Conclusions

We report the genotypic and phenotypic spectra of 20 Chinese patients with BBS. The clinical phenotype of Chinese patients with BBS was similar to that of the international cohort; however, some differences were identified. We addressed the association between the genotype and the phenotype of patients with BBS to assess prognosis based on the causative mutation. Patients with *BBS2, BBS7,* and *BBS9* gene variants had more severe visual symptoms and multiple vision problems. Patients with variations in *BBS10*, and *BBS12* had an earlier age of onset and diagnosis, which may partly reflect their poor prognosis. These findings provide new insights into the biology of the BBSome complex and the roles of the individual *BBS* genes in humans.

For fetuses with polydactyly, genitourinary abnormalities, and/or hydrometrocolpos, BBS should be considered. Additionally, early diagnosis is crucial, which could improve the prognosis of these patients, preventing some chronic damage, such as renal insufficiency, after an early surgical correction of urological abnormalities. In addition, continuous follow-up of these patients is expected to be valuable, as the clinical features among individuals are generally variable and diverse. Moreover, other features, including renal dysfunction and cognitive impairment, affect both the long-term prognosis and quality of life in patients with BBS, highlighting the necessity of collecting a comprehensive database of ciliopathy phenotypes to make progress in the diagnosis and treatment of BBS.

### Supplementary Information

**Supplementary Material 1.**

## Data Availability

The datasets generated and/or analyzed during the current study are not publicly available but are available from the corresponding author on reasonable request.

## Abbreviations

BBS: Bardet-Biedl Syndrome
BMI: Body mass index
LOF: Loss-of-function
ACMG: The American College of Medical Genetics and Genomics
F: Female
M: Male
RD: Retinal dystrophy
PD: Polydactyly
CI: Cognitive impairment
RA: Renal abnormalities
T2DM: Type 2 diabetes mellitus
UA: Urogenital anomalies
OD: Ovarian dysplasia
EGD: External genital dysplasia
OC: Ovarian cyst
UD: Uterine hypopiasia
VA: Vaginal atresia
RC: Renal cyst
CKD: Chronic kidney dysfunction
