# Supplementary material for: Molecular and phenotypic characteristics of Bardet-Biedl syndrome in Chinese patients
Source: Orphanet J Rare Dis. 2024 Apr 8;19:149. doi: 10.1186/s13023-024-03150-9 (PMC11000329; doi:10.1186/s13023-024-03150-9)
Supplement: Supplementary file 1 — Supplementary Material 1. [file 13023_2024_3150_MOESM1_ESM.docx]

**Supplementary Table 1** 196 inherited retinal disease genes analyzed by targeted NGS diagnostic testing.

| *ABCA4* | *BZRAP1* | *DRAM2* | *INVS* | *MYO5B* | *PRPF8* | *SPP2* |
| --- | --- | --- | --- | --- | --- | --- |
| *ABCB6* | *C1QTNF5* | *EFEMP1* | *IQCB1* | *MYO7A* | *PRPH2* | *TCTN2* |
| *ABCC6* | *C2orf71* | *ELOVL4* | *ITGB1* | *NEK1* | *PTHB1* | *TIMP3* |
| *ACAD11* | *C5orf42* | *EMC1* | *KCNJ13* | *NEK2* | *RBP3* | *TMEM67* |
| *ADIPOQ* | *C8orf37* | *ENO2* | *KCNV2* | *NEK8* | *RD3* | *TNK2* |
| *ADIPOR1* | *CA4* | *EYS* | *KIAA1549* | *NEUROD1* | *RDH5* | *TOPORS* |
| *AGBL5* | *CCDC28B* | *FAM161A* | *KIF15* | *NMNAT1* | *RDH12* | *TRIM32* |
| *AHI1* | *CEP164* | *FHAD1* | *KIF5A* | *NPHP1* | *REEP6* | *TRNT1* |
| *AIPL1* | *CEP290* | *FSCN2* | *KIF7* | *NPHP3* | *RGR* | *TTC21B* |
| *ALMS1* | *CEP83* | *FTO* | *KIFC3* | *NPHP4* | *RHO* | *TTC8* |
| *ANKS6* | *CERKL* | *GCOM1* | *KIZ* | *NR2E3* | *RLBP1* | *TULP1* |
| *ARHGEF18* | *CFH* | *GDF6* | *KLHL7* | *NRL* | *ROM1* | *UBD* |
| *ARL2BP* | *CHM* | *GLI2* | *LCA5* | *NUB1* | *RP1* | *USH1C* |
| *ARL3* | *CLCC1* | *GLIS2* | *LCA5L* | *NXNL1* | *RP1L1* | *USH1G* |
| *ARL6* | *CLRN1* | *GNAT1* | *LEP* | *OFD1* | *RP2* | *USH2A* |
| *ATXN10* | *CNGA1* | *GPR125* | *LRAT* | *OTX2* | *RP9* | *VCAN* |
| *BAZ2B* | *CNGA3* | *GUCA1B* | *LRRFIP2* | *PDC* | *RPE65* | *VPS13B* |
| *BBIP1* | *CNGB1* | *GUCY2D* | *LZTFL1* | *PDE6B* | *RPGR* | *WDPCP* |
| *BBS1* | *CNGB3* | *HGSNAT* | *MAK* | *PDE6G* | *RPGRIP1* | *XPNPEP3* |
| *BBS10* | *COMT* | *HK1* | *MAOA* | *PHLDB1* | *RPGRIP1L* | *ZNF408* |
| *BBS12* | *CRB1* | *HMCN1* | *MECP2* | *POMGNT1* | *SAG* | *ZNF423* |
| *BBS2* | *CRX* | *IDH3B* | *MEHMO* | *PPFIA2* | *SAMD11* | *ZNF513* |
| *BBS20* | *CTNNA1* | *IFT140* | *MERTK* | *PRCD* | *SDCCAG8* |  |
| *BBS4* | *CUX1* | *IFT172* | *MFSD8* | *PRDM13* | *SEMA4A* |  |
| *BBS5* | *CYP4V2* | *IFT27* | *MKKS* | *PROM1* | *SHH* |  |
| *BBS7* | *DCDC2* | *IMPDH1* | *MKS1* | *PRPF3* | *SLC41A1* |  |
| *BBS9* | *DHDDS* | *IMPG1* | *MRE11A,* | *PRPF31* | *SLC7A14* |  |
| *BDNF* | *DISC1* | *IMPG2* | *MVK* | *PRPF4* | *SNRNP200* |  |
| *BEST1* | *DHX38* | *INPP5E* | *MYH9* | *PRPF6* | *SPATA7* |  |

**Supplementary Table 2** Systemic manifestations of patients with BBS in this study.

| **Patient** | **Gender** | **Age(y)** | **Age at onset(m)** | **Age at diagnosis(y)** | **Initial symptom** | **RD** | **OB** | **PD** | **CI** | **Gonad** | **RA** | **Tooth** | **Hear** | **Smell** | **Heart** | **Liver** | **Neurology** | **Development delay** | **Diabetes** | **Others** |
| --- | --- | --- | --- | --- | --- | --- | --- | --- | --- | --- | --- | --- | --- | --- | --- | --- | --- | --- | --- | --- |
| F1 | F | 11 | 12 | 8 | vision decline | Y | N | 2hands and 2 feet | Y | N | N | N | N | Y | N | N | N | Y | N |  |
| F2 | F | 17 | 1 | 14 | overweight | Y | Y | 2hands and 2 feet | N | OD | N | N | N | N | NA | Raised ALT | N | Y | T2DM | hyperuricemia、hypothyroidism |
| F3 | M | 13 | 3 | 13 | Night blindness | Y | Y | 2 feet | Y | EGD | RC | NA | NA | NA | N | NAFLD | NA | Y | N | hypospadias |
| F4 | M | 21 | 12 | 15 | overweight | Y | Y | 1hand and 2 feet | Y | EGD | N | N | N | N | N | N | N | Y | N | hyperuricemia |
| F5 | M | 26 | 120 | 25 | Night blindness | Y | Y | 2 feet | N | EGD | NA | hypodontia | N | N | NA | NAFLD | N | N | insulin resistance |  |
| F6 | M | 28 | 72 | 10 | Night blindness | Y | Y | 2 feet | N | EGD | N | N | N | N | N | N | N | N | N |  |
| F7 | F | 30 | 3 | 7 | Night blindness | Y | Y | 2 feet | Y | OC | N | N | N | N | NA | N | N | Y | N |  |
| F8 | M | 32 | 12 | 32 | Night blindness | Y | Y | Right hand and left foot | N | N | N | microdontia | N | Y | N | N | N | N | N |  |
| F9 | F | 34 | 24 | 12 | Night blindness | Y | Y | 1hand and 2 feet | N | N | NA | hypodontia | N | N | NA | N | N | Y | insulin resistance |  |
| F10 | F | 12 | 4 | 7 | over weight | Y | Y | 2hands and 2 feet | Y | N | RC | microdontia | N | N | NA | N | N | Y | N | fetuses with polydactyly, |
| F11 | M | 11 | 18 | 10 | Night blindness | Y | Y | 2hands and 2 feet | Y | EGD | N | microdontia | N | N | N | N | epilepsy | Y | N |  |
| F12 | M | 10 | 36 | 5 | Night blindness | Y | Y | Left foot | N | EGD | N | N | N | N | N | N | N | Y | N |  |
| F13 | F | 11 | 48 | 4 | vision decline | Y | N | Right hand and foot | N | UD | N | N | N | N | N | N | N | Y | N | Imperforate anus  fetuses with polydactyly |
| F14 | M | 9 | 24 | 7 | Night blindness | Y | Y | 2hands and 2 feet | Y | EGD | N | N | N | N | N | NAFLD | N | Y | T2DM | Hyperuricemia,fetuses with polydactyly |
| F15 | M | 8 | Since birth | 1 | Night blindness | Y | Y | 2hands and 2 feet | Y | EGD | RC、CKD | hypodontia | N | N | N | N | N | Y | N | Congenital megacolon，  Recurrent urinarytractinfections |
| F16 | F | 9 | Since birth | 0.17 | Night blindness | Y | Y | Right hand | N | VA | RC | microdontia | N | N | N | N | N | Y | insulin resistance |  |
| F17-II | F | 5 | 3 | Since birth | vision decline | N | Y | Left foot | N | N | N | N | N | N | N | N | N | N | N |  |
| F17-I | M | 12 | 4 | 10 | overweight | Y | Y | N | N | EGD | N | microdontia | N | N | N | N | N | N | N |  |
| F18-II | M | 7 | 3 | 6 | Night blindness | Y | No | 2hands and 2 feet | Y | EGS | N | N | N | N | N | N | epilepsy | Y | N |  |
| F18-I | F | 14 | 24 | 9 | Night blindness | Y | Y | 2hands and 2 feet | Y | OC | N | N | N | N | N | N | epilepsy | Y | N |  |
| Total | 9F/11M |  |  |  |  | 19/20 | 17/20 | 19/20 | 10/20 | 15/20 | 4/18 | 8/19 | 0/19 | 2/19 | 0/15 | 4/20 | 3/19 | 15/20 | 5/20 |  |

F, female; M, male; Y, yes; N, no; NA, data not available; RD, retinal dystrophy; PD, polydactyly; CI, cognitive impairment; RA, renal abnormalities; T2DM, type 2 diabetes mellitus; UA, urogenital anomalies; OD, ovarian dysplasia; EGD, external genital dysplasia; OC, ovarian cyst; UD, uterine hypopiasia; VA, vaginal atresia; RC, renal cyst; CKD, chronic kidney dysfunction; NAFLD, nonalcoholic fatty liver disease; ALT, alanine aminotransfease; OB/OW, obesity or over weight: defined as BMI ≥28 kg/m^2^ or BMI ≥24 kg/m^2^ for adults according to the WGOC criteria. For children between 6 and 18 years old, overweight/obesity was defined according to Chinese reference values released by the National Health and Family Planning Commission of the People’s Republic of China. For patients aged 5 years, obesity was defined as a BMI-for-age greater than two SD, above the WHO, growth reference median; and overweight was defined as a BMI-for-age greater greater than one SD, above the WHO Growth Reference median.

**Supplementary Table 3** Summary of BBS variants in 85 Chinese BBS families (18 families in this study and 67 families in previous reports).

| **Gene** | **Nucleotide change** | **Amino acid change** | **No. Of alleles** | **References** |
| --- | --- | --- | --- | --- |
| BBS1 | c.260-261del | p.A87Lfs*64 | 1 | Li et al. (2017)^[48]^ |
| BBS1 | c.421C>T | p.Q141X | 2 | Qian et al. (2019) ^[49]^ |
| BBS1 | c.1045G>C | p.G349R(p.Gly349Arg) | 1 | Tang et al. (2022)^[50]^ |
| BBS1 | c.1177C>T | p.Arg393* | 2 | Cai et al. (2022)^[51]^ |
| BBS1 | c.1339G>A | p.A447T | 1 | Li et al. (2017)^[48]^ |
| BBS1 | c.1376_c.1377insGCGCCTACGTGCTGCCC | p.L459Lfs*23(p.Leu459Leufs*23) | 1 | Tang et al. (2022)^[50]^ |
| BBS2 | c.235A>C | p.Thr79Pro | 1 | This study |
| BBS2 | c.289C>T | p.Gln97* | 1 | This study |
| BBS2 | c.943C>T | p.R315W | 1 | This study |
| BBS2 | c.47C>T | p.Pro16Leu | 1 | Gao et al. (2019)^[39]^ |
| BBS2 | c.235T > G | p.T79P | 1 | Huang et al. (2021)^[52]^ |
| BBS2 | c.646C>T | p.R216X | 1 | Ding et al. (2016)^[53]^ |
| BBS2 | c.685T>A | p.Y229X | 1 | Li et al. (2017)^[48]^ |
| BBS2 | c.779(exon7)T>G | p.L260R | 1 | Tang et al. (2022)^[50]^ |
| BBS2 | c.844A>T | p.Lys282Ter | 1 | Hu et al. (2014)^[54]^ |
| BBS2 | c.944G > A | p.R315Q | 1 | Meng et al. (2021)^[41]^ |
| BBS2 | c.1015C > T | p.R339* | 1 | Meng et al. (2021)^[41]^ |
| BBS2 | c.1206dupA | p.R403fs | 1 | Ding et al. (2016)^[53]^ |
| BBS2 | c.1237C>T | p.Arg413* | 1 | Dan et al. (2020) ^[55]^ |
| BBS2 | c.1278A > G | p.E426E | 1 | Meng et al. (2021)^[41]^ |
| BBS2 | c.1398-1G>A |  | 1 | Tang et al. (2022)^[49]^ |
| BBS2 | c.1797G>A | p.Lys599Lys | 1 | Gao et al. (2019)^[39]^ |
| BBS2 | c.1910+2_+3insT |  | 1 | Gao et al. (2019)^[39]^ |
| BBS2 | c.2059 + 1G > T |  | 1 | Meng et al. (2021)^[41]^ |
| BBS2 | c.2059 + 1G > C |  | 1 | Meng et al. (2021)^[41]^ |
| BBS2 | c.79A>C | p.Thr27Pro | 4 | This Study  Meng et al. (2021)^[41]^ |
| BBS2 | c.534+1G>T |  | 10 | This Study  Wu et al. (2022) ^[22]^  Tao et al. (2022) ^[56]^ Meng et al. (2021)^[41]^  Huang et al. (2021)^[52]^  Zhong et al. (2023) ^[57]^ |
| BBS2 | c.563del | p.Ile188Thrfs*13 | 5 | This Study  Dan et al. (2020) ^[55]^  Xing et al. (2014) ^[58]^  Zhong et al. (2023) ^[57]^ |
| BBS2 | c.685T>C | p.Tyr229His | 2 | This Study  Gao et al. (2019)^[39]^ |
| BBS2 | c.700C>T | p.Arg234* | 4 | This Study  Gao et al. (2019)^[39]^ |
| BBS2 | c.1148-1149dupTC | p.His384Serfs*34 | 2 | This study,  Chen et al. (2017) ^[23]^ |
| BBS2 | c.1438C>T | p.Arg480Ter | 4 | This Study  Hu et al. (2014)^[54]^ Xing et al. (2014) ^[58]^ |
| BBS2 | c.2107C>T | p.Arg703Ter | 2 | This Study  Gao et al. (2019)^[39]^ |
| BBS3/ARL6 | c.364C>T | p.R122X | 2 | Xing et al. (2014) ^[58]^ |
| BBS4 | c.31C > T | p.Q11* | 1 | Meng et al. (2021)^[41]^ |
| BBS4 | c.70A>T | p.K24X | 6 | Li et al. (2017)^[48]^  Li et al. (2014) ^[59]^ |
| BBS4 | c.932G > A | p.G311D | 1 | Meng et al. (2021)^[41]^ |
| BBS4 | c.1106+2T>A |  | 2 | Li et al. (2022) ^[60]^ |
| BBS5 | c.1A>G | p.Met1Val | 1 | Shao et al. (2022) ^[61]^ |
| BBS5 | c.613C>T | p.Q205* | 2 | Tao et al. (2022) ^[56]^ |
| BBS6/MKKS | c.635C>T | p.Ser212Phe | 1 | Li et al. (2022) ^[62]^ |
| BBS6/MKKS | c.748G > A | p.G250R | 2 | Huang et al. (2021)^[52]^ |
| BBS6/MKKS | c.1175C>T | p.T392M | 1 | Qi et a. (2017 )^[63]^ |
| BBS6/MKKS | c.1192C>T | p.Q398* | 1 | Qi et a. (2017 )^[63]^ |
| BBS6/MKKS | c.1496G>A | p.C499Y | 2 | Xing et al. (2014) ^[58]^ |
| BBS6/MKKS | c.1664C>G | p.Thr555Arg | 1 | Li et al. (2022) ^[62]^ |
| BBS7 | c.288_289delAG | p.G97Kfs*7 | 5 | Li et al. (2017)^[48]^,  Tao et al. (2020) ^[64]^ |
| BBS7 | c.389_390delAC | p.Asn130fs | 6 | Yao et al. (2017) ^[65]^,  Xiu et al. (2018) ^[66]^,  Shen et al. (2019)^[67]^ |
| BBS7 | c.1002del | p.Asn335fs* | 7 | Li et al. (2017)^[48]^  Tao et al. (2020) ^[64]^  Jing et al. (2021) ^[68]^ |
| BBS7 | c.1666A>C | p.S556R | 4 | Liu et al. (2008) ^[69]^  Yang et al. (2008)^[70]^ |
| BBS7 | c.728G>A | p.C243Y | 4 | This study,  Li et al. (2017)^[48]^,  Meng et al. (2021)^[41]^ |
| BBS7 | c.1395T > A | p.Y465* | 2 | This study,  Li et al. (2017)^[48]^  ^]^ |
| BBS9 | c.460A>T | p.lle154Phe | 1 | This study |
| BBS9 | c.1215-1216del | p.E408Rfs*1 | 1 | This study |
| BBS9 | c.1561C>T | p.Arg521Ter | 1 | This study |
| BBS9 | c.1789+1G>T |  | 1 | This study |
| BBS9 | c.72delT | p.L25Cfs*16 | 1 | Meng et al. (2021)^[41]^ |
| BBS9 | c.263 + 2delT |  | 1 | Meng et al. (2021)^[41]^ |
| BBS9 | c.433delG | p.Gly145Valfs2 | 1 | Gao et al. (2019)^[39]^ |
| BBS9 | c.445C > T | p.R149* | 3 | Meng et al. (2021)^[41]^  Tang et al 2021 ^[71]^ |
| BBS9 | c.1114C>T | p.Q372X | 2 | Zhang et al. (2021)^[72]^ |
| BBS9 | c.1198 + 1G > A |  | 1 | Meng et al. (2021)^[41]^ |
| BBS9 | c.1759C>T | p.Arg587Ter | 2 | Gao et al. (2019)^[39]^ |
| BBS10 | c.145C>T | p.Arg49Trp | 1 | This study |
| BBS10 | c.980C>T | p.G1y327Va1 | 1 | This study |
| BBS10 | c.1130G>A | p.Arg377Lys | 1 | This study |
| BBS10 | c.1514-1520del | p.Pro505fs | 1 | This study |
| BBS10 | c.2093T>G | p.Ile698Arg | 1 | This study |
| BBS10 | c.378G>A | p.W126* | 1 | Tao et al. (2020) ^[64]^ |
| BBS10 | c.445_446insC | p.L149Pfs*3 | 2 | Lin et al. (2018)^[24]^,  This study |
| BBS10 | c.539G>A | p.G180E | 6 | Wang et al. (2018) ^[73]^  Li et al. (2022) ^[60]^  Tao et al. (2022) ^[56]^，  Tao et al. (2020) ^[64]^ |
| BBS10 | c.602G>A | p.C201Y | 2 | Wang et al. (2018) ^[73]^  Tao et al. (2020) ^[64]^ |
| BBS10 | c.784_785delGA | p.Glu262fs* | 1 | Dong et al. (2022) ^[74]^ |
| BBS10 | c.891_897delinsTTTGT | p.Met298LeufsTer5 | 2 | Liu et al. (2021) ^[75]^ |
| BBS10 | c.1063C>T | p.Q355* | 1 | Tao et al. (2020) ^[64]^ |
| BBS10 | c.1812dupT | p.Asn605* | 1 | Dong et al. (2022) ^[74]^ |
| BBS10 | c.1391C>G | p.S464* | 3 | This study  Tao et al. (2022) ^[56]^ |
| BBS12 | c.590_591del | p.Tyr197fs | 2 | This study |
| BBS12 | c.1124_1125del | p.Ser375fs | 2 | This study |
| BBS12 | c.1649T>C | p.Leu550Pro | 2 | This study |
| BBS12 | c.155delC | p.S52* | 1 | Tao et al. (2022) ^[56]^ |
| BBS12 | c.173delA | p.E58Efs*5 | 2 | Zhai et al. (2018) ^[76]^  Tang et al. (2022)^[50]^ |
| BBS12 | c.188delC | p.T63fs | 1 | Shen et al. (2022) ^[77]^ |
| BBS12 | c.1276T>C | p.C426R | 2 | Tao et al. (2020) ^[64]^ |
| BBS12 | c.1584T>G | p.Y528* | 1 | Tao et al. (2022) ^[56]^ |
| BBS12 | c.1604T>G | p.V535G | 1 | Zhai et al. (2018) ^[76]^  Tang et al. (2022)^[50]^ |
| BBS12 | c.1749(exon3)_c.1750(exon3)ins |  | 1 | Tang et al.2022^[50]^ |
| BBS12 | c.1749_1750delA | p.R584Dfs*54 | 1 | Zhai et al. (2018) ^[76]^ |
| BBS12 | c.1783T>C | p.W595R | 1 | Zhai et al. (2018) ^[76]^  Tang et al. (2022)^[50]^ |
| BBS12 | c.1993_1995del | p.665_665del | 1 | Shen et al. (2022) ^[77]^ |
| BBS12 | c.1320_1326dupTGTGATG | p.Gin443fs | 4 | This study，  Li et al. (2022) ^[60]^ |
| BBS13/MKS1 | c.1382A>G | p.Y461C | 1 | Xing et al. (2014) ^[58]^ |
| BBS13/MKS1 | c.1601G>A | p.R534Q | 1 | Xing et al. (2014) ^[58]^ |
| BBS16 | c.845(exon8)_c.848(exon8)ins |  | 2 | Tang et al. (2022)^[50]^ |
| CEP290 | c.7328_7332dup | p.V2445Rfs*3 | 1 | Zhu et al. (2022) ^[78]^ |
| CEP290 | c.4708C>T | p.Q1570* | 1 | Zhu et al. (2022) ^[78]^ |
